# Supplementary figures and images for: Organisation and delivery of a dedicated multidisciplinary prone ventilation team in the intensive care unit: Strategies and lessons from COVID-19
Source: PLoS One. 2023 Dec 28;18(12):e0296379. doi: 10.1371/journal.pone.0296379 (PMC10754430; doi:10.1371/journal.pone.0296379)

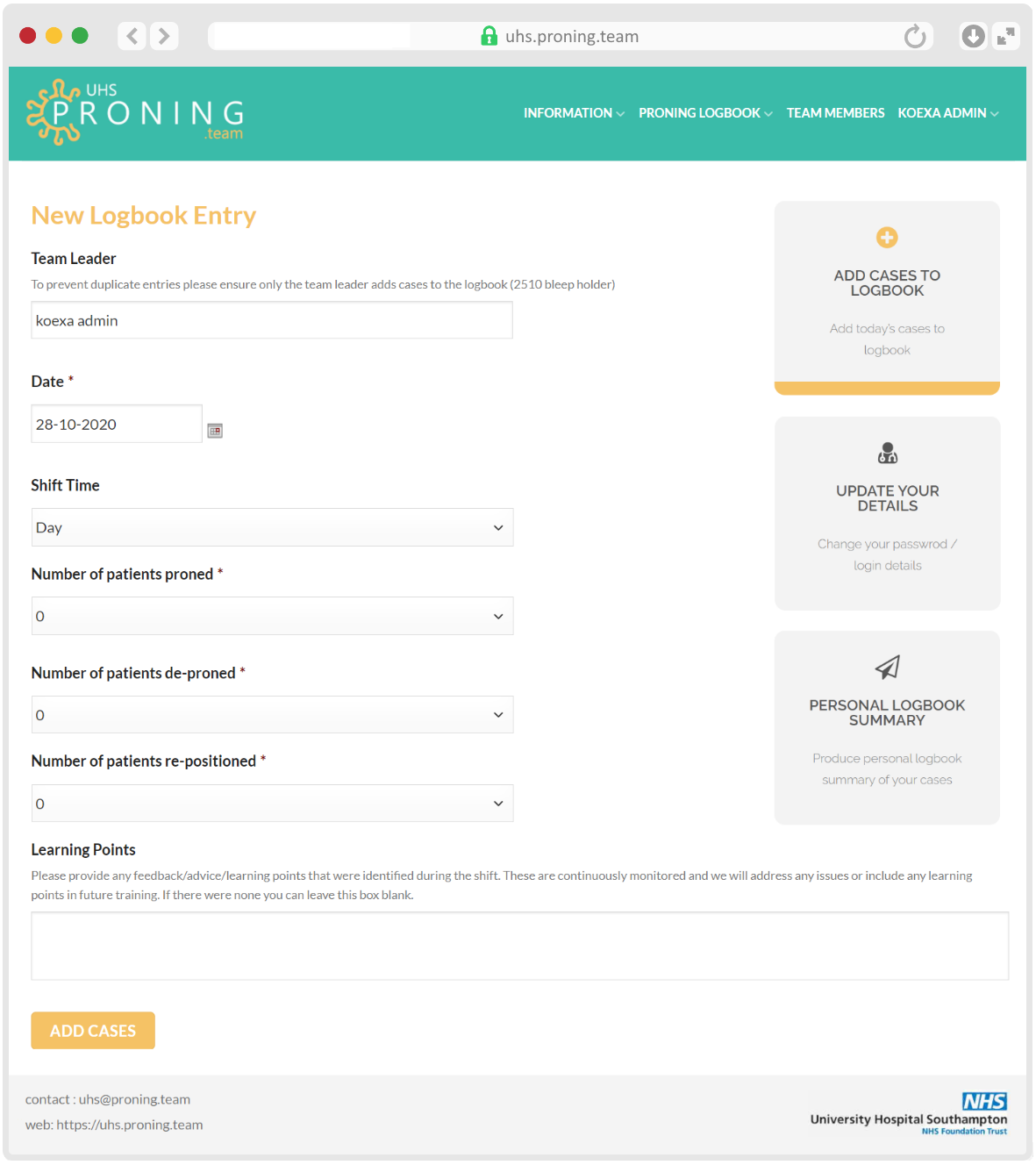

Supplement: S1 Appendix — (TIFF) [file pone.0296379.s001.tiff]

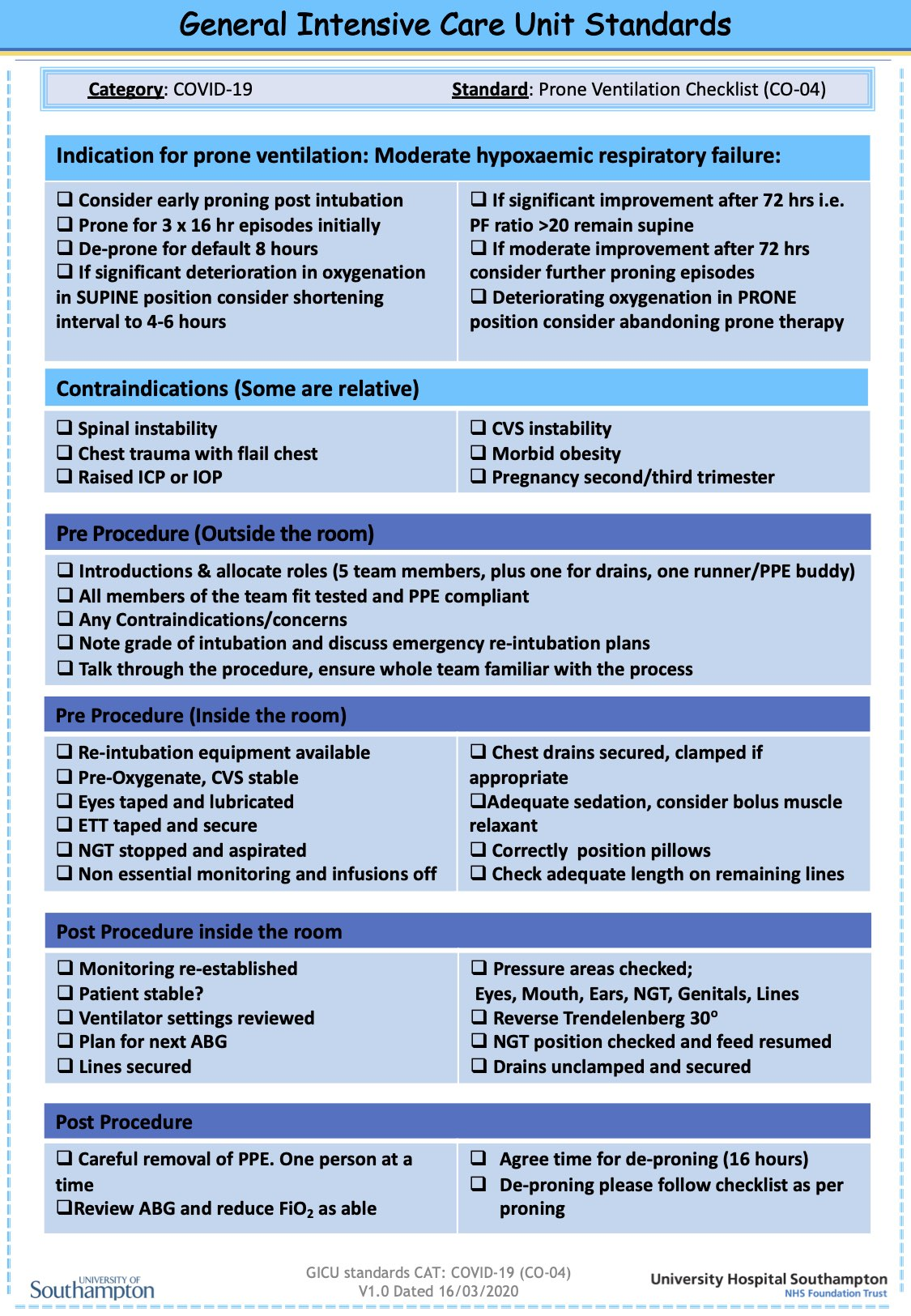

Supplement: S2 Appendix — (TIFF) [file pone.0296379.s002.tiff]
