# Supplementary material for: Organisation and delivery of a dedicated multidisciplinary prone ventilation team in the intensive care unit: Strategies and lessons from COVID-19
Source: PLoS One. 2023 Dec 28;18(12):e0296379. doi: 10.1371/journal.pone.0296379 (PMC10754430; doi:10.1371/journal.pone.0296379)
Supplement: S1 File — (DOCX) [file pone.0296379.s003.docx]

Q1 What is your age?

Answered: 61

Skipped: 0

18-24

25-34

35-44

45-54

55-64

65+

0

%

10

%

20

%

30

%

40

%

50

%

60

%

70

%

80

%

90

%

100

%

| **ANSWER CHOICES** | **RESPONSES** |  |
| --- | --- | --- |
| 18-24 | 9.84% | 6 |
| 25-34 | 40.98% | 25 |
| 35-44 | 22.95% | 14 |
| 45-54 | 22.95% | 14 |
| 55-64 | 3.28% | 2 |
| 65+ | 0.00% | 0 |
| TOTAL |  | 61 |

Q2 What is your gender?

Answered: 61 Skipped: 0

|  |  |  |  |  |  |  |  |  |  |
| --- | --- | --- | --- | --- | --- | --- | --- | --- | --- |

Female

Male

Prefer not to say

0% 10% 20% 30% 40% 50% 60% 70% 80% 90% 100%

| **ANSWER CHOICES** | **RESPONSES** |  |
| --- | --- | --- |
| Female | 80.33% | 49 |
| Male | 19.67% | 12 |
| Prefer not to say | 0.00% | 0 |
| TOTAL |  | 61 |

Q3 What is your normal Job Role?

Answered: 61

Skipped: 0

Senior Doctor

)

Consultant

(

Trainee Doctor

Nurse normally

works in ICU...

Nurse normally

works in acu...

Nurse normally

works in OPD...

ODP

Other theatre

staff

Allied Health

Care...

Health Care

Assistants

Non clinical

Other (please

specify)

%

0

%

10

%

20

%

30

40

%

%

50

60

%

70

%

80

%

90

%

100

%

| **ANSWER CHOICES** | **RESPONSES** |  |
| --- | --- | --- |
| Senior Doctor (Consultant) | 4.92% | 3 |
| Trainee Doctor | 19.67% | 12 |
| Nurse normally works in ICU environment | 0.00% | 0 |
| Nurse normally works in acute setting | 27.87% | 17 |
| Nurse normally works in OPD setting | 6.56% | 4 |
| ODP | 4.92% | 3 |
| Other theatre staff | 3.28% | 2 |
| Allied Health Care Professional | 0.00% | 0 |
| Health Care Assistants | 14.75% | 9 |
| Non clinical | 0.00% | 0 |
| Other (please specify) | 18.03% | 11 |
| TOTAL |  | 61 |

| **#** | **OTHER (PLEASE SPECIFY)** | **DATE** |
| --- | --- | --- |
| 1 | Nurse, normally working in research | 7/7/2020 12:47 PM |
| 2 | specialist nurse | 7/5/2020 7:58 AM |
| 3 | Student Nurse Apprentice | 7/4/2020 1:35 AM |
| 4 | NP in surgical pre assessment | 7/3/2020 12:44 PM |
| 5 | Research Physiotherapist | 6/29/2020 12:37 PM |
| 6 | Practice educator for theatres/ Recovery nurse | 6/29/2020 9:31 AM |
| 7 | Clinical trials assistant | 6/29/2020 9:08 AM |
| 8 | Clinical Trials Assistant | 6/24/2020 1:38 PM |
| 9 | Tissue Viability Nurse | 6/24/2020 7:49 AM |
| 10 | Tissue viability | 6/24/2020 7:38 AM |
| 11 | Student nurse | 6/23/2020 6:02 PM |

Q4 Did you specifically volunteer for a role on the proning team?

Answered: 61 Skipped: 0

|  |  |  |  |  |  |  |  |  |  |
| --- | --- | --- | --- | --- | --- | --- | --- | --- | --- |

Yes

No

0% 10% 20% 30% 40% 50% 60% 70% 80% 90% 100%

| **ANSWER CHOICES** | **RESPONSES** |  |
| --- | --- | --- |
| Yes | 37.70% | 23 |
| No | 62.30% | 38 |
| TOTAL |  | 61 |

Q5 How anxious were you about the possibility of joining the proning team?

Answered: 61

Skipped: 0

Not anxious

Mildly anxious

Moderately

anxious

Very anxious

Severely

anxious

%

0

%

10

20

%

%

30

40

%

50

%

60

%

70

%

%

80

90

%

100

%

| **ANSWER CHOICES** | **RESPONSES** |  |
| --- | --- | --- |
| Not anxious | 22.95% | 14 |
| Mildly anxious | 39.34% | 24 |
| Moderately anxious | 22.95% | 14 |
| Very anxious | 9.84% | 6 |
| Severely anxious | 3.28% | 2 |
| TOTAL |  | 61 |

| **#** | **ANY COMMENT** | **DATE** |
| --- | --- | --- |
| 1 | Worried about disconnection/extubation | 7/8/2020 5:01 PM |
| 2 | just at first | 7/7/2020 2:54 PM |
| 3 | I did not join the team, I was working on GICU | 7/7/2020 12:47 PM |
| 4 | Prone regularly in ICU | 7/7/2020 2:27 AM |
| 5 | I was most anxious about the part when you had to 'turn' the patient onto their front | 7/4/2020 1:35 AM |
| 6 | never had any ITU experience. just back on phased return after arm injury. As we sit within critical care , it was suggested that we would stay within that foot print rather than going to other areas that i would have been more comfortable to go too (T&O) | 7/3/2020 12:44 PM |
| 7 | the expectations of the critical care team from us | 7/2/2020 11:41 AM |
| 8 | I was mainly axious about the unknown | 6/29/2020 5:56 PM |
| 9 | Not anxious about actual proning but concerned about working with COVID + patients | 6/26/2020 3:52 PM |
| 10 | I don’t know what I’m going to go through , in the end it was so rewarding to be part of the proning team | 6/24/2020 2:56 PM |
| 11 | My only 'anxiety' was with regards to joining a new team/role as opposed to any 'anxiety' regarding the work or setting itself. | 6/24/2020 1:38 PM |
| 12 | we are used to seeing patients in ITU but did feel anxious about working with a team of new people and learning a new skill | 6/24/2020 7:38 AM |
| 13 | i was moderately anxious because it was a new thing to me. | 6/23/2020 7:42 PM |
| 14 | New staff, from outpatients at Lymington Hospital moving to the proning team at uhs during a global pandemic! | 6/23/2020 7:06 PM |
| 15 | But working alongside others, I felt supported and happy to be helping make a difference. | 6/23/2020 6:04 PM |

Q6 Did you have any specific concerns about joining the proning team?

Please select all that apply

Answered: 61

Skipped: 0

Yes, Managing

very sick...

Yes, Infection

risk to myself

Yes, Infection

risk to my...

Yes,

Unfamiliar...

Yes, Never

done this...

Yes, Due to

antisocial...

No concerns

Other (please

specify)

%

0

%

10

%

20

30

%

40

%

50

%

%

60

70

%

80

%

90

%

100

%

| **ANSWER CHOICES** | | **RESPONSES** | |  |
| --- | --- | --- | --- | --- |
| Yes, Managing very sick patients | | 34.43% | | 21 |
| Yes, Infection risk to myself | | 50.82% | | 31 |
| Yes, Infection risk to my family and close contacts | | 47.54% | | 29 |
| Yes, Unfamiliar environment | | 44.26% | | 27 |
| Yes, Never done this before | | 47.54% | | 29 |
| Yes, Due to antisocial hours/shift work | | 9.84% | | 6 |
| No concerns | | 11.48% | | 7 |
| Other (please specify) | | 6.56% | | 4 |
| Total Respondents: 61 | |  | |  |
| **#** | **OTHER (PLEASE SPECIFY)** | | **DATE** | |
| 1 | Not applicable | | 7/7/2020 12:47 PM | |
| 2 | Taken away from usual clinical work, physically hard work | | 7/6/2020 10:52 AM | |
| 3 | I had just returned from mat leave and I am from BAME background | | 6/29/2020 12:37 PM | |
| 4 | Having not worked nights for over 10 years I was concerned how I would manage. This proved manageable | | 6/26/2020 3:52 PM | |

Q7 Did you receive formal training on how to prone and reposition patients? If no, please skip to Question 19.

Answered: 61 Skipped: 0

|  |  |  |  |  |  |  |  |  |  |
| --- | --- | --- | --- | --- | --- | --- | --- | --- | --- |

Yes

No

0% 10% 20% 30% 40% 50% 60% 70% 80% 90% 100%

| **ANSWER CHOICES** | **RESPONSES** |  |
| --- | --- | --- |
| Yes | 91.80% | 56 |
| No | 8.20% | 5 |
| TOTAL |  | 61 |

Q8 How well do you think the training prepared you?

Answered: 54

Skipped: 7

A great deal

A lot

A moderate

amount

A little

None at all

%

0

%

10

20

%

30

%

40

%

50

%

60

%

70

%

80

%

90

%

100

%

| **ANSWER CHOICES** | **RESPONSES** |  |
| --- | --- | --- |
| A great deal | 14.81% | 8 |
| A lot | 40.74% | 22 |
| A moderate amount | 25.93% | 14 |
| A little | 18.52% | 10 |
| None at all | 0.00% | 0 |
| TOTAL |  | 54 |

Q9 Do you feel that you had enough training/ practice?

Answered: 54 Skipped: 7

|  |  |  |  |  |  |  |  |  |  |
| --- | --- | --- | --- | --- | --- | --- | --- | --- | --- |

Yes

No

0% 10% 20% 30% 40% 50% 60% 70% 80% 90% 100%

| **ANSWER CHOICES** | **RESPONSES** |  |
| --- | --- | --- |
| Yes | 77.78% | 42 |
| No | 22.22% | 12 |
| TOTAL |  | 54 |

Q10 Were you able to practice physically proning a person or mannequin during this training?

Answered: 55 Skipped: 6

|  |  |  |  |  |  |  |  |  |  |
| --- | --- | --- | --- | --- | --- | --- | --- | --- | --- |

Yes

No

0% 10% 20% 30% 40% 50% 60% 70% 80% 90% 100%

| **ANSWER CHOICES** | **RESPONSES** |  |
| --- | --- | --- |
| Yes | 83.64% | 46 |
| No | 16.36% | 9 |
| TOTAL |  | 55 |

Q11 Were you shown and encouraged to use the safety checklist for proning?

Answered: 57 Skipped: 4

|  |  |  |  |  |  |  |  |  |  |
| --- | --- | --- | --- | --- | --- | --- | --- | --- | --- |

Yes

No

0% 10% 20% 30% 40% 50% 60% 70% 80% 90% 100%

| **ANSWER CHOICES** | **RESPONSES** |  |
| --- | --- | --- |
| Yes | 82.46% | 47 |
| No | 17.54% | 10 |
| TOTAL |  | 57 |

Q12 Were you made aware of the proning team resources website (proning.team)?

Answered: 56 Skipped: 5

|  |  |  |  |  |  |  |  |  |  |
| --- | --- | --- | --- | --- | --- | --- | --- | --- | --- |

Yes

No

0% 10% 20% 30% 40% 50% 60% 70% 80% 90% 100%

| **ANSWER CHOICES** | **RESPONSES** |  |
| --- | --- | --- |
| Yes | 26.79% | 15 |
| No | 73.21% | 41 |
| TOTAL |  | 56 |

Q13 If so, did you find this website useful?

Answered: 54

Skipped: 7

Extremely

useful

Very useful

Somewhat useful

Not so useful

Not at all

useful

I was not

aware of the...

%

0

%

10

20

%

30

%

%

40

50

%

60

%

70

%

80

%

%

90

100

%

| **ANSWER CHOICES** | **RESPONSES** |  |
| --- | --- | --- |
| Extremely useful | 9.26% | 5 |
| Very useful | 12.96% | 7 |
| Somewhat useful | 5.56% | 3 |
| Not so useful | 1.85% | 1 |
| Not at all useful | 0.00% | 0 |
| I was not aware of the website | 70.37% | 38 |
| TOTAL |  | 54 |

Q14 Were you made aware of the proning team training video?

Answered: 56 Skipped: 5

|  |  |  |  |  |  |  |  |  |  |
| --- | --- | --- | --- | --- | --- | --- | --- | --- | --- |

Yes

No

0% 10% 20% 30% 40% 50% 60% 70% 80% 90% 100%

| **ANSWER CHOICES** | **RESPONSES** |  |
| --- | --- | --- |
| Yes | 39.29% | 22 |
| No | 60.71% | 34 |
| TOTAL |  | 56 |

Q15 If so, did you find this video useful?

Answered: 54

Skipped: 7

Extremely

useful

Very useful

Somewhat useful

Not so useful

Not at all

useful

I was not

aware of thi...

%

0

%

10

%

20

30

%

%

40

50

%

60

%

70

%

80

%

%

90

100

%

| **ANSWER CHOICES** | **RESPONSES** |  |
| --- | --- | --- |
| Extremely useful | 9.26% | 5 |
| Very useful | 25.93% | 14 |
| Somewhat useful | 5.56% | 3 |
| Not so useful | 0.00% | 0 |
| Not at all useful | 0.00% | 0 |
| I was not aware of this video | 59.26% | 32 |
| TOTAL |  | 54 |

Q16 Is there anything else you think would have been useful to include in the training that wasn't?

Answered: 23 Skipped: 38

| **#** | **RESPONSES** | **DATE** |
| --- | --- | --- |
| 1 | More sessions for sho s in ITU | 7/12/2020 11:06 AM |
| 2 | None | 7/6/2020 11:13 PM |
| 3 | No | 7/6/2020 8:43 PM |
| 4 | No. | 7/4/2020 1:06 AM |
| 5 | No | 7/3/2020 11:33 PM |
| 6 | There was many refresher updates put on by the trust in the lead up to this pandemic and were encouraged to attend. I was kept updated on a regular basis about UHS and our care group which was useful. Although I don't remember being given specific web sites to look at about proning I did undertake to look at these myself, which were very useful. I was given information about Apps for ITU, care for ventilated patients , and hand outs and proning check list | 7/3/2020 12:44 PM |
| 7 | no | 7/3/2020 11:53 AM |
| 8 | the proning video on the proning resources was introduced at a later date and was not accessed by all members of the proning team. The checklist was not available during the training session in theatre | 7/2/2020 11:41 AM |
| 9 | If there was ever another pandemic that required a proning team I would be happy to support | 6/29/2020 5:56 PM |
| 10 | I wasn't made aware of the training sessions. I just happened to find out. While the training session was good, the communication wasn't (and I completely understand it was a chaotic period) and I wasn't aware of the training website and video. That would've helped tremendously. | 6/29/2020 12:37 PM |
| 11 | No, good practical session | 6/29/2020 8:38 AM |
| 12 | I think the use of the mannequin in training did not truly reflect the size of the patients requiring proning. | 6/26/2020 3:52 PM |
| 13 | n/a | 6/25/2020 4:15 AM |
| 14 | Preparation mentally/physically especially for those who never been exposed in critical setting . I would suggest as not every staff volunteer to be part of this would be beneficial esp. for the first timer . Advance briefing prior maybe useful . Also not everyone has been aware of the web / video it was only after that I found out about it. I’m grateful I have been part of the team though sadly I only done it once . The training & experienced made very useful to me too . | 6/24/2020 2:56 PM |
| 15 | The process was all very systematic and simple so I felt the training was definitely sufficient | 6/24/2020 1:38 PM |
| 16 | No | 6/24/2020 11:47 AM |
| 17 | Proning different sizes and weights of patients, having lines attached to the mannequin to better simulate a realistic patient setup | 6/24/2020 8:37 AM |
| 18 | No | 6/24/2020 7:49 AM |
| 19 | Had training very early on during Covid outbreak therefore video may not have been available yet. | 6/23/2020 9:21 PM |
| 20 | non | 6/23/2020 7:42 PM |
| 21 | I felt much better about the whole situation after the training so it was good. I think if I had been called in to prone I would have wanted to run through it all again as proning a mannequin in a theatre would be very different to doing it in full ppe, on real people in a busy ICU. As it turns out I haven’t been required... so far but I am it would be good to practice with donning and doffing and maybe with a real (volunteer for practice) person. | 6/23/2020 7:06 PM |
| 22 | Watching the video would have been helpful. I helped prone only a couple of times at the beginning, so perhaps the video was available after this. | 6/23/2020 6:04 PM |
| 23 | N.a | 6/23/2020 6:02 PM |

Q17 Following training, did you subsequently join the proning team on

GICU? If yes, please continue with the rest of the survey. If no, please ignore the rest of the survey and scroll to the bottom and click "Done".

Thank you for your participation.

Answered: 54

Skipped: 7

Yes

No, I wasn't

rostered to...

No, I didn't

want to

0

%

10

%

%

20

30

%

40

%

%

50

60

%

70

%

%

80

90

%

100

%

| **ANSWER CHOICES** | **RESPONSES** |  |
| --- | --- | --- |
| Yes | 57.41% | 31 |
| No, I wasn't rostered to work there | 42.59% | 23 |
| No, I didn't want to | 0.00% | 0 |
| TOTAL |  | 54 |

Q18 Did you have the opportunity to undertake a mentored/supernumerary shift with the proning team?

Answered: 48

Skipped: 13

Yes, and I

found this...

Yes, but it

wasn't helpful

No, but I

don't think ...

No, but I

would have...

Other (please

specify)

%

0

%

10

20

%

30

%

40

%

50

%

60

%

70

%

80

%

%

90

100

%

| **ANSWER CHOICES** | | **RESPONSES** | |  |
| --- | --- | --- | --- | --- |
| Yes, and I found this helpful | | 16.67% | | 8 |
| Yes, but it wasn't helpful | | 0.00% | | 0 |
| No, but I don't think it would have been helpful | | 50.00% | | 24 |
| No, but I would have liked one | | 20.83% | | 10 |
| Other (please specify) | | 12.50% | | 6 |
| TOTAL | |  | | 48 |
| **#** | **OTHER (PLEASE SPECIFY)** | | **DATE** | |
| 1 | No. as I have other Clinical role | | 7/7/2020 2:54 PM | |
| 2 | Not applicable | | 7/7/2020 12:47 PM | |
| 3 | No | | 7/6/2020 8:43 PM | |
| 4 | Worked in ICU so not needed to join the proning team | | 7/6/2020 8:33 AM | |
| 5 | was put on a coordinator role and it was the first time that I had to physically position patient | | 7/2/2020 11:41 AM | |
| 6 | I was told I will be in proning team a day before my shift | | 6/24/2020 2:56 PM | |

Q19 What were your normal roles on the proning team?

Answered: 45

Skipped: 16

Airway

Team leader

Turning patient

Fine

positioning

Assistant

Other (please

specify)

%

0

%

10

20

%

30

%

40

%

50

%

60

%

70

%

80

%

90

%

100

%

| **ANSWER CHOICES** | | **RESPONSES** |  | |  |
| --- | --- | --- | --- | --- | --- |
| Airway | | 31.11% |  | | 14 |
| Team leader | | 42.22% |  | | 19 |
| Turning patient | | 64.44% |  | | 29 |
| Fine positioning | | 28.89% |  | | 13 |
| Assistant | | 28.89% |  | | 13 |
| Other (please specify) | | 11.11% |  | | 5 |
| Total Respondents: 45 | |  |  | |  |
| **#** | **OTHER (PLEASE SPECIFY)** | | | **DATE** | |
| 1 | Not on team | | | 7/7/2020 12:47 PM | |
| 2 | Not airway, but turning pt, and working towards being team leader | | | 7/3/2020 12:44 PM | |
| 3 | airway support for the anaesthetist | | | 7/2/2020 11:41 AM | |
| 4 | going through the checklist, assess tissue | | | 6/29/2020 12:37 PM | |
| 5 | I only had to be a team leader on one occasion due to staffing issues on one night, but it wasn't an issue. | | | 6/24/2020 1:38 PM | |

Q20 Roughly how many proning shifts have you done?

Answered: 52

Skipped: 9

No shifts

shifts

<10

10-20

shifts

21-30

shifts

31-40

shifts

shifts

>40

%

0

%

10

%

20

30

%

40

%

%

50

60

%

70

%

80

%

90

%

%

100

| **ANSWER CHOICES** | **RESPONSES** |  |
| --- | --- | --- |
| No shifts | 19.23% | 10 |
| <10 shifts | 38.46% | 20 |
| 10-20 shifts | 26.92% | 14 |
| 21-30 shifts | 9.62% | 5 |
| 31-40 shifts | 3.85% | 2 |
| >40 shifts | 1.92% | 1 |
| TOTAL |  | 52 |

Q21 Considering an average shift, how heavy was the work load?

Answered: 43

Skipped: 18

Very light

Light

Neither light

nor heavy

Heavy

Very heavy

Not sure

%

0

%

10

20

%

%

30

40

%

50

%

60

%

70

%

%

80

90

%

100

%

| **ANSWER CHOICES** | **RESPONSES** |  |
| --- | --- | --- |
| Very light | 0.00% | 0 |
| Light | 0.00% | 0 |
| Neither light nor heavy | 20.93% | 9 |
| Heavy | 65.12% | 28 |
| Very heavy | 6.98% | 3 |
| Not sure | 6.98% | 3 |
| TOTAL |  | 43 |

Q22 To what extent would you agree that there was good team work within the proning team?

Answered: 44

Skipped: 17

Strongly agree

Agree

Neither agree

nor disagree

Disagree

Strongly

disagree

%

0

%

10

20

%

%

30

40

%

50

%

60

%

70

%

%

80

90

%

100

%

| **ANSWER CHOICES** | **RESPONSES** |  |  |
| --- | --- | --- | --- |
| Strongly agree | 70.45% |  | 31 |
| Agree | 22.73% |  | 10 |
| Neither agree nor disagree | 4.55% |  | 2 |
| Disagree | 0.00% |  | 0 |
| Strongly disagree | 2.27% |  | 1 |
| TOTAL |  |  | 44 |

Q23 To what extent would you agree that there was good teamwork between the ICU team and the proning team?

Answered: 45

Skipped: 16

Strongly agree

Agree

Somewhat agree

Neither agree

nor disagree

Somewhat

disagree

Disagree

Strongly

disagree

%

0

%

10

20

%

30

%

40

%

50

%

60

%

70

%

80

%

90

%

100

%

| **ANSWER CHOICES** | **RESPONSES** |  |  |
| --- | --- | --- | --- |
| Strongly agree | 35.56% |  | 16 |
| Agree | 35.56% |  | 16 |
| Somewhat agree | 13.33% |  | 6 |
| Neither agree nor disagree | 6.67% |  | 3 |
| Somewhat disagree | 6.67% |  | 3 |
| Disagree | 2.22% |  | 1 |
| Strongly disagree | 0.00% |  | 0 |
| TOTAL |  |  | 45 |

Q24 To what extent would you agree that there was good teamwork between the anaesthetic members and the proning team?

Answered: 44 Skipped: 17

|  |  |  |  |  |  |  |  |  |  |
| --- | --- | --- | --- | --- | --- | --- | --- | --- | --- |
|  |  |  |  |  |  |  |  |  |  |
|  |  |  |  |  |  |  |  |  |  |

Strongly agree

Agree

Somewhat agree

Neither agree nor disagree

Somewhat disagree Disagree

Strongly disagree

0% 10% 20% 30% 40% 50% 60% 70% 80% 90% 100%

| **ANSWER CHOICES** | **RESPONSES** |  |  |
| --- | --- | --- | --- |
| Strongly agree | 50.00% |  | 22 |
| Agree | 47.73% |  | 21 |
| Somewhat agree | 0.00% |  | 0 |
| Neither agree nor disagree | 2.27% |  | 1 |
| Somewhat disagree | 0.00% |  | 0 |
| Disagree | 0.00% |  | 0 |
| Strongly disagree | 0.00% |  | 0 |
| TOTAL |  |  | 44 |

Q25 To what extent would you agree with the following statement: I was always aware of who was leading the proning team

Answered: 44

Skipped: 17

Strongly agree

Agree

Somewhat agree

Neither agree

nor disagree

Somewhat

disagree

Disagree

Strongly

disagree

%

0

%

10

%

20

30

%

40

%

50

%

60

%

70

%

80

%

90

%

100

%

| **ANSWER CHOICES** | **RESPONSES** |  |  |
| --- | --- | --- | --- |
| Strongly agree | 65.91% |  | 29 |
| Agree | 29.55% |  | 13 |
| Somewhat agree | 0.00% |  | 0 |
| Neither agree nor disagree | 4.55% |  | 2 |
| Somewhat disagree | 0.00% |  | 0 |
| Disagree | 0.00% |  | 0 |
| Strongly disagree | 0.00% |  | 0 |
| TOTAL |  |  | 44 |

Q26 To what extent would you agree with the following: I knew how/who/where to escalate any issues or concerns

Answered: 44

Skipped: 17

Strongly agree

Agree

Neither agree

nor disagree

Disagree

Strongly

disagree

%

0

%

10

20

%

%

30

40

%

50

%

60

%

70

%

%

80

90

%

100

%

| **ANSWER CHOICES** | **RESPONSES** |  |  |
| --- | --- | --- | --- |
| Strongly agree | 43.18% |  | 19 |
| Agree | 27.27% |  | 12 |
| Neither agree nor disagree | 15.91% |  | 7 |
| Disagree | 11.36% |  | 5 |
| Strongly disagree | 2.27% |  | 1 |
| TOTAL |  |  | 44 |

Q27 To what extent would you agree with the following:I felt confident that any issues or concerns I raised would be taken seriously and

addressed accordingly

Answered: 44

Skipped: 17

Strongly agree

Agree

Neither agree

nor disagree

Disagree

Strongly

disagree

%

0

%

10

20

%

%

30

40

%

50

%

60

%

70

%

%

80

90

%

100

%

| **ANSWER CHOICES** | **RESPONSES** |  |
| --- | --- | --- |
| Strongly agree | 34.09% | 15 |
| Agree | 36.36% | 16 |
| Neither agree nor disagree | 22.73% | 10 |
| Disagree | 4.55% | 2 |
| Strongly disagree | 2.27% | 1 |
| TOTAL |  | 44 |

Q28 To what extent would you agree with the following: I felt valued as part of the proning team.

Answered: 44

Skipped: 17

Strongly agree

Agree

Somewhat agree

Neither agree

nor disagree

Somewhat

disagree

Disagree

Strongly

disagree

%

0

%

10

20

%

30

%

40

%

50

%

60

%

70

%

80

%

90

%

100

%

| **ANSWER CHOICES** | **RESPONSES** |  |  |
| --- | --- | --- | --- |
| Strongly agree | 47.73% |  | 21 |
| Agree | 34.09% |  | 15 |
| Somewhat agree | 2.27% |  | 1 |
| Neither agree nor disagree | 11.36% |  | 5 |
| Somewhat disagree | 0.00% |  | 0 |
| Disagree | 2.27% |  | 1 |
| Strongly disagree | 2.27% |  | 1 |
| TOTAL |  |  | 44 |

Q29 How often did you witness an emergency or clinical problem during a proning event?

Answered: 44

Skipped: 17

Very often

Often

Sometimes

Rarely

Never

0

%

10

%

%

20

30

%

40

%

%

50

60

%

70

%

%

80

90

%

100

%

| **ANSWER CHOICES** | **RESPONSES** |  |
| --- | --- | --- |
| Very often | 0.00% | 0 |
| Often | 0.00% | 0 |
| Sometimes | 34.09% | 15 |
| Rarely | 43.18% | 19 |
| Never | 22.73% | 10 |
| TOTAL |  | 44 |

Q30 In your experience, what emergencies/problems occurred most frequently during a proning manoeuvre?

Answered: 44

Skipped: 17

Airway

emergencies

Ventilation/cir

cuit issues

Cardiovascular

emergencies

Loss of lines,

tubes etc

No

emergencies/...

%

0

%

10

20

%

30

%

40

%

50

%

60

%

70

%

80

%

%

90

100

%

| **ANSWER CHOICES** | | **RESPONSES** | |  |
| --- | --- | --- | --- | --- |
| Airway emergencies | | 4.55% | | 2 |
| Ventilation/circuit issues | | 43.18% | | 19 |
| Cardiovascular emergencies | | 9.09% | | 4 |
| Loss of lines, tubes etc | | 9.09% | | 4 |
| No emergencies/problems occurred | | 31.82% | | 14 |
| TOTAL | |  | | 44 |
| **#** | **OTHER (PLEASE SPECIFY)** | | **DATE** | |
| 1 | My patient stopped ventilating, I was at the airway end | | 7/12/2020 11:06 AM | |
| 2 | Disconnection x1 | | 7/8/2020 5:01 PM | |
| 3 | occ cardiovascular emergencies | | 7/3/2020 12:44 PM | |
| 4 | Re-intubation of an ASA 4 and witnessed about 5xcircuit disconnections. However, with the use of proning checklist it was avoided | | 7/2/2020 11:41 AM | |
| 5 | inadequate sedation, cardiovascular istability | | 6/29/2020 12:37 PM | |
| 6 | during my 9 shifts we had 4 vent tubing disconnections and one reintubation emergency | | 6/29/2020 9:31 AM | |
| 7 | Can't comment | | 6/23/2020 7:03 PM | |

Q31 How frequently did you use the proning check list?

Answered: 44 Skipped: 17

|  |  |  |  |  |  |  |  |  |  |
| --- | --- | --- | --- | --- | --- | --- | --- | --- | --- |

Always

Usually

Sometimes

Rarely

Never

0% 10% 20% 30% 40% 50% 60% 70% 80% 90% 100%

| **ANSWER CHOICES** | **RESPONSES** |  |
| --- | --- | --- |
| Always | 70.45% | 31 |
| Usually | 22.73% | 10 |
| Sometimes | 2.27% | 1 |
| Rarely | 2.27% | 1 |
| Never | 2.27% | 1 |
| TOTAL |  | 44 |

Q32 Did you find the proning check list useful?

Answered: 45 Skipped: 16

|  |  |  |  |  |  |  |  |  |  |
| --- | --- | --- | --- | --- | --- | --- | --- | --- | --- |

Yes

No

Not sure

0% 10% 20% 30% 40% 50% 60% 70% 80% 90% 100%

| **ANSWER CHOICES** | **RESPONSES** |  |
| --- | --- | --- |
| Yes | 93.33% | 42 |
| No | 2.22% | 1 |
| Not sure | 4.44% | 2 |
| TOTAL |  | 45 |

Q33 Would you add any additional check points to the checklist?

Answered: 45

Skipped: 16

Yes

No

Not sure

0

%

%

10

20

%

%

30

40

%

50

%

60

%

%

70

80

%

90

%

100

%

| **ANSWER CHOICES** | | **RESPONSES** |  |  | |  |
| --- | --- | --- | --- | --- | --- | --- |
| Yes | | 6.67% |  |  | | 3 |
| No | | 60.00% |  |  | | 27 |
| Not sure | | 31.11% |  |  | | 14 |
| TOTAL | |  |  |  | | 45 |
| **#** | **IF YES, WHAT WOULD YOU ADD?** | | | | **DATE** | |
| 1 | Grade of intubation | | | | 7/12/2020 10:47 AM | |
| 2 | not enough experience | | | | 7/7/2020 2:54 PM | |
| 3 | Check all connections in breathing circuit | | | | 7/7/2020 2:27 AM | |
| 4 | 1 memeber of staff didnt always use the proning check list, and i felt that i couldnt challenge that at the beginning , so i discussed with my team leader and suggested that if we also had a laminated check list on the airway trolley then we always had one to refer too, and also had spares kept with the proning turn board | | | | 7/3/2020 12:44 PM | |
| 5 | Maybe lead person being acknowledged before starting | | | | 6/24/2020 11:47 AM | |

Q34 Was there a printed proning checklist available at every bed space?

Answered: 43

Skipped: 18

Always

Usually

Sometimes

Rarely

Never

0

%

%

10

20

%

30

%

%

40

50

%

60

%

70

%

%

80

90

%

100

%

| **ANSWER CHOICES** | **RESPONSES** |  |
| --- | --- | --- |
| Always | 18.60% | 8 |
| Usually | 46.51% | 20 |
| Sometimes | 23.26% | 10 |
| Rarely | 9.30% | 4 |
| Never | 2.33% | 1 |
| TOTAL |  | 43 |

Q35 Do you think the use of proning checklist prevented any patient safety incidents?

Answered: 45

Skipped: 16

Yes

No

Not sure

0

%

%

10

20

%

%

30

40

%

50

%

60

%

%

70

80

%

90

%

100

%

| **ANSWER CHOICES** | **RESPONSES** |  |
| --- | --- | --- |
| Yes | 91.11% | 41 |
| No | 4.44% | 2 |
| Not sure | 4.44% | 2 |
| TOTAL |  | 45 |

Q36 Were you involved in any patient safety incidents that you believe the use of the checklist may have prevented?

Answered: 44

Skipped: 17

Yes

No

Not sure

0

%

%

10

20

%

%

30

40

%

50

%

60

%

%

70

80

%

90

%

100

%

| **ANSWER CHOICES** | | **RESPONSES** |  |  | |  |
| --- | --- | --- | --- | --- | --- | --- |
| Yes | | 9.09% |  |  | | 4 |
| No | | 84.09% |  |  | | 37 |
| Not sure | | 6.82% |  |  | | 3 |
| TOTAL | |  |  |  | | 44 |
| **#** | **IF YES, PLEASE COMMENT** | | | | **DATE** | |
| 1 | re-intubation of ASA 4 | | | | 7/2/2020 11:41 AM | |
| 2 | The vent tubing disconnections, the check list explictly says to check the connection although Drs did not always complete this check | | | | 6/29/2020 9:31 AM | |
| 3 | displacement of the arterial line. Inadvertant extubation due to ventilaotr tubing being trapped in bedspace | | | | 6/26/2020 3:52 PM | |
| 4 | Clear escalation process with contact numbers and clear identification of who is responsible that day for adressing concerns | | | | 6/23/2020 7:03 PM | |

Q37 To what extent would you agree with the following statement:I had access to appropriate PPE for every proning shift

Answered: 44

Skipped: 17

Strongly agree

Agree

Neither agree

nor disagree

Disagree

Strongly

disagree

%

0

%

10

20

%

%

30

40

%

50

%

60

%

70

%

%

80

90

%

100

%

| **ANSWER CHOICES** | **RESPONSES** |  |  |
| --- | --- | --- | --- |
| Strongly agree | 59.09% |  | 26 |
| Agree | 20.45% |  | 9 |
| Neither agree nor disagree | 9.09% |  | 4 |
| Disagree | 11.36% |  | 5 |
| Strongly disagree | 0.00% |  | 0 |
| TOTAL |  |  | 44 |

Q38 Did you spend longer than 4 hours in PPE at any one time?

Answered: 45

Skipped: 16

Always

Usually

Sometimes

Rarely

Never

0

%

10

%

%

20

30

%

40

%

%

50

60

%

70

%

%

80

90

%

100

%

| **ANSWER CHOICES** | **RESPONSES** |  |
| --- | --- | --- |
| Always | 0.00% | 0 |
| Usually | 4.44% | 2 |
| Sometimes | 24.44% | 11 |
| Rarely | 33.33% | 15 |
| Never | 37.78% | 17 |
| TOTAL |  | 45 |

Q39 In general, did you feel that you had sufficient breaks while on shifts?

Answered: 44

Skipped: 17

Always

Usually

Sometimes

Rarely

Never

0

%

10

%

%

20

30

%

40

%

%

50

60

%

70

%

%

80

90

%

100

%

| **ANSWER CHOICES** | **RESPONSES** |  |
| --- | --- | --- |
| Always | 50.00% | 22 |
| Usually | 34.09% | 15 |
| Sometimes | 15.91% | 7 |
| Rarely | 0.00% | 0 |
| Never | 0.00% | 0 |
| TOTAL |  | 44 |

Q40 In general, did you feel well rested between shifts?

Answered: 44

Skipped: 17

Always

Usually

Sometimes

Rarely

Never

0

%

%

10

20

%

30

%

%

40

50

%

60

%

70

%

%

80

90

%

100

%

| **ANSWER CHOICES** | **RESPONSES** |  |
| --- | --- | --- |
| Always | 22.73% | 10 |
| Usually | 45.45% | 20 |
| Sometimes | 25.00% | 11 |
| Rarely | 4.55% | 2 |
| Never | 2.27% | 1 |
| TOTAL |  | 44 |

Q41 Did you suffer any symptoms consistent with COVID-19 infection during your time with the proning team?

Answered: 44

Skipped: 17

Yes, but I

wasn't tested

Yes, but I

tested negative

Yes, and I

tested positive

No

%

0

%

10

20

%

%

30

40

%

50

%

60

%

70

%

%

80

90

%

100

%

| **ANSWER CHOICES** | | **RESPONSES** |  | |  |
| --- | --- | --- | --- | --- | --- |
| Yes, but I wasn't tested | | 2.27% |  | | 1 |
| Yes, but I tested negative | | 4.55% |  | | 2 |
| Yes, and I tested positive | | 6.82% |  | | 3 |
| No | | 86.36% |  | | 38 |
| TOTAL | |  |  | | 44 |
| **#** | **ADDITIONAL COMMENTS** | | | **DATE** | |
| 1 | I hgad symptoms on april 16th, self isolated and tested positive on the 24th of april. | | | 6/29/2020 9:31 AM | |
| 2 | This was later after I finished proning | | | 6/24/2020 12:01 PM | |

Q42 Did you suffer any physical injury as a result of working with the proning team?

Answered: 44 Skipped: 17

|  |  |  |  |  |  |  |  |  |  |
| --- | --- | --- | --- | --- | --- | --- | --- | --- | --- |

Yes

No

0% 10% 20% 30% 40% 50% 60% 70% 80% 90% 100%

| **ANSWER CHOICES** | | **RESPONSES** |  |  | |  |
| --- | --- | --- | --- | --- | --- | --- |
| Yes | | 6.82% |  |  | | 3 |
| No | | 93.18% |  |  | | 41 |
| TOTAL | |  |  |  | | 44 |
| **#** | **IF YES, PLEASE COMMENT** | | | | **DATE** | |
| 1 | Not sure if it exacabated my knee symptoms.Have seen consultant, now wating knee scope/debride | | | | 7/3/2020 12:44 PM | |
| 2 | during the first 2 shifts, had some muscle aches after the shift | | | | 7/2/2020 11:41 AM | |
| 3 | Not injury but sore muscles till I got used to the work | | | | 6/29/2020 12:37 PM | |
| 4 | Some back ache even though I used moving and handling techniques | | | | 6/24/2020 12:01 PM | |
| 5 | Not from proning as didnt prone but yes from prolonged duration in PPE now have two scars from face mask | | | | 6/23/2020 7:03 PM | |

Q43 To what extent would you say your psychological health was negatively affected as a result of your work with the proning team?

Answered: 44

Skipped: 17

Not at all

Slightly

Moderately

Significantly

0

%

10

%

20

%

30

%

40

%

50

%

60

%

70

%

80

%

90

%

%

100

| **ANSWER CHOICES** | **RESPONSES** |  |
| --- | --- | --- |
| Not at all | 45.45% | 20 |
| Slightly | 43.18% | 19 |
| Moderately | 9.09% | 4 |
| Significantly | 2.27% | 1 |
| TOTAL |  | 44 |

Q44 Did you need to take any time out of work due to any of the above issues?

Answered: 44 Skipped: 17

|  |  |  |  |  |  |  |  |  |  |
| --- | --- | --- | --- | --- | --- | --- | --- | --- | --- |

Yes

No

0% 10% 20% 30% 40% 50% 60% 70% 80% 90% 100%

| **ANSWER CHOICES** | | **RESPONSES** |  | |  |  |
| --- | --- | --- | --- | --- | --- | --- |
| Yes | | 9.09% |  | |  | 4 |
| No | | 90.91% |  | |  | 40 |
| TOTAL | |  |  | |  | 44 |
| **#** | **IF YES, PLEASE SPECIFY HOW LONG** | | | **DATE** | | |
| 1 | 5 days due to covid infection | | | 7/12/2020 10:47 AM | | |
| 2 | But it was very hard to prone memebers of staff that i used to work with. I was made aware of their admission by the team leader before i went in to ITU. During quiet periods i returned back to my dept, i did experience some animosity , which was rather upsetting, which i had to report to my manager. | | | 7/3/2020 12:44 PM | | |
| 3 | When i tested postiive I had 13 days off. | | | 6/29/2020 9:31 AM | | |
| 4 | I was ill the following day after my shift with the proning team . But I prior to my proning shift I was moved to CICU then moved the same day to surveillance ward for the afternoon shift . I was off for 4 weeks then back to work become unwell so off work extended for another 2 weeks . | | | 6/24/2020 2:56 PM | | |
| 5 | After leaving ITU had a weeks annual leave to get over not being in PPE | | | 6/23/2020 7:03 PM | | |

Q45 To what extent do you agree with the following: I felt proud of my role on the proning team

Answered: 43

Skipped: 18

Strongly agree

Agree

Neither agree

nor disagree

Disagree

Strongly

disagree

%

0

%

10

20

%

%

30

40

%

50

%

60

%

70

%

80

%

%

90

100

%

| **ANSWER CHOICES** | **RESPONSES** |  |  |
| --- | --- | --- | --- |
| Strongly agree | 58.14% |  | 25 |
| Agree | 30.23% |  | 13 |
| Neither agree nor disagree | 11.63% |  | 5 |
| Disagree | 0.00% |  | 0 |
| Strongly disagree | 0.00% |  | 0 |
| TOTAL |  |  | 43 |

Q46 To what extent do you agree with the following:I would be happy to join the proning team again

Answered: 43

Skipped: 18

Strongly agree

Agree

Neither agree

nor disagree

Disagree

Strongly

disagree

%

0

%

10

20

%

%

30

40

%

50

%

60

%

70

%

%

80

90

%

100

%

| **ANSWER CHOICES** | **RESPONSES** |  |  |
| --- | --- | --- | --- |
| Strongly agree | 69.77% |  | 30 |
| Agree | 23.26% |  | 10 |
| Neither agree nor disagree | 4.65% |  | 2 |
| Disagree | 2.33% |  | 1 |
| Strongly disagree | 0.00% |  | 0 |
| TOTAL |  |  | 43 |

Q47 Overall, how did you feel about being part of the proning team?

Answered: 42

Skipped: 19

Very positive

Positive

Neutral

Negative

Very negative

0

%

10

%

%

20

30

%

40

%

%

50

60

%

70

%

%

80

90

%

100

%

| **ANSWER CHOICES** | **RESPONSES** |  |
| --- | --- | --- |
| Very positive | 61.90% | 26 |
| Positive | 30.95% | 13 |
| Neutral | 7.14% | 3 |
| Negative | 0.00% | 0 |
| Very negative | 0.00% | 0 |
| TOTAL |  | 42 |

Q48 Can you think of any changes that could be made to improve the proning service for patients and staff?

Answered: 24 Skipped: 37

| **#** | **RESPONSES** | **DATE** |
| --- | --- | --- |
| 1 | Anaesthetists asked to become additional ITU SpR at one stage rather than dedicated to proning. This didn’t seem well thought though at all as the proning team was really busy and needed a full time anaesthetist on the team. This was introduced on a bank holiday with no notice and I think no consultation with anaesthetists. Many of us felt undervalued by this. Within a few days this plan had failed and we were back to proning...which most of us would have predicted would happen and we would have said if we had been consulted! Other than that it was run very well. | 7/12/2020 10:47 AM |
| 2 | Start of shift brief/team meet and decipher experience and roles. | 7/8/2020 5:01 PM |
| 3 | More consistency in the teams + team members in each team | 7/7/2020 2:27 AM |
| 4 | None I could think of. But if includes the area where we do the proning, I wish the area is a bit cooler. It is already hot enough wearing full PPE, and if we enter the area that is also hot, it feels more suffocating to be inside. | 7/6/2020 11:13 PM |
| 5 | Please never ask us to wear body bags again - the most demoralising moment of my whole career | 7/6/2020 11:10 AM |
| 6 | Workload much harder OOH. Would be useful to split the shift into 2 so each Anaesthethist does 6 hours each depending on other work | 7/6/2020 10:52 AM |
| 7 | Communication with the ITU doctors was challenging. Often didn’t know where they were and we sometimes had mixed messages about whether they wanted us to prone/deprone patients. Also the ITU nurses were quite obstructive sometimes (Particularly about paralysis) and occasionally even misrepresented the truth to discourage me from giving muscle relaxant. | 7/6/2020 10:51 AM |
| 8 | For training staff to be aware of the checklist and video | 7/4/2020 1:35 AM |
| 9 | No | 7/3/2020 11:33 PM |
| 10 | no | 7/3/2020 12:44 PM |
| 11 | Introduction of the team and meeting the whole staff involved so as to have a quick overview of what the role entails and to go through the process of the roles and responsibilities. Also, to ensure that everyone has had the appropriate training required. | 7/2/2020 11:41 AM |
| 12 | Given that the entire service was set up in peak pandemic, it was excellent but looking into the future I would suggest we could improve the organisation, training, communication, mental health support. | 6/29/2020 12:37 PM |
| 13 | Better roster organisation- perhaps delegating this task to a few seniors. | 6/29/2020 9:31 AM |
| 14 | clearer links with ITU to identify which patients required proning | 6/29/2020 8:38 AM |
| 15 | A meeting with the dedicated airway person at the start of shift to discuss plan for each patient regarding timings for turning. I felt at times , the patients care had to depend on the anaesthetists availability rather than best practice. | 6/26/2020 3:52 PM |
| 16 | n/a | 6/25/2020 4:15 AM |
| 17 | I think the proning service is great & really done amazing job. Though individual readiness & preparations I think plays a big part . Training with a dummy is easier than doing it in real patient . To add in the training a full video of proning patient I think is good idea. Because not all staff who went to the dummy training in proning have been aware of the proning video website. Another improvement is briefing session prior to a shift start . If you are a first timer you don’t really know what to expect . | 6/24/2020 2:56 PM |
| 18 | Although not an issue with the proning service itself, it would have been better if PPE wasn't so short so we were able to complete turns/swims based on patient schedules/needs as opposed to trying to conserve PPE as best we can. | 6/24/2020 1:38 PM |
| 19 | Enough Proning checklist and PPE | 6/24/2020 12:01 PM |
| 20 | Personally I would have liked to have known more about the patient for example an update on how they were clinically progressing or not progressing. | 6/24/2020 11:47 AM |
| 21 | Nothing specifically about service delivery, but sometimes organising teams was a little difficult id staff were allocated to finish their shift during a proning session. | 6/24/2020 7:49 AM |
| 22 | Think the proning team and many in ITU did an amazing job | 6/23/2020 7:03 PM |

23 I enjoyed the great team working approach on the proning team. Sometimes there was a 6/23/2020 6:04 PM

|  | little tension with ITU staff this could be improved to make everyone feel valued |  |
| --- | --- | --- |
| 24 | No changes, the team was amazing. | 6/23/2020 6:02 PM |

Q49 Any other comments?

Answered: 21 Skipped: 40

| **#** | **RESPONSES** | **DATE** |
| --- | --- | --- |
| 1 | I believe that formal recognition by the critical care department and/or the trust of those members of staff who contributed to the proning teams (especially those new to critical care or from non-clinical backgrounds) would be a very positive step considering the arduous nature of the work and the positive impact it had on patient outcomes. | 7/12/2020 9:46 PM |
| 2 | See above | 7/12/2020 10:47 AM |
| 3 | I was on the proning bleep later in the pandemic spike when the bleep was also the helper ICU reg. So my views are perhaps skewed a little. I did not know who my proning team were as they were no longer meeting in the mornings and I wasn't sure how to get hold of them. The ICU nurses largely became the team and I did on one occasion feel resistance to me using the checklist as if I was slowing things up for them. I was also challenged when I asked for rocuronium to paralyse the patient. | 7/8/2020 5:01 PM |
| 4 | I worked in GICU with patients that were attended by the proning team. IN the vast majority of cases it worked really, really well. The day that they were not allowed in because of PPE shortages, they were really missed. My colleagues in London hospitals had very different experiences of proning teams from me. They did not use the checklist and had a number of safety incidents | 7/7/2020 12:47 PM |
| 5 | no | 7/7/2020 2:27 AM |
| 6 | Proning teams were working really hard, sometimes hard to then watch groups of ICU doctors just sitting...…. | 7/6/2020 11:10 AM |
| 7 | The tissue viability nurses were EXCELLENT at organising the teams and turns. They were so resourceful and organised even out of their usual environments. They made the shifts so much easier as they took on pretty much all the mental load with organising the rounds | 7/6/2020 10:52 AM |
| 8 | I'm very proud of the work we've done and the relationships we've built. | 7/5/2020 7:58 AM |
| 9 | No | 7/3/2020 11:33 PM |
| 10 | How well UHS adapted and put these measures in place to manage covid patients. | 7/3/2020 12:44 PM |
| 11 | I felt proud of being part of the team. | 7/2/2020 11:41 AM |
| 12 | As discussed above, coming from BAME background, my mental health really suffered as it was just around the time reports of deaths of healthcare professionals started to emerge. As we were several new staff joining the team in the beginning, I would've felt better prepared if I was given some briefing/ pep talk. The leader didn't check what levels of training we had or explain how the shift was expected to go. We were expected to don, go inside, follow, observe and learn- which is fine but prior communication would've helped tremendously for new starters. A couple of team leaders insisted that we turn patients every 3 hours and we used up a lot of PPE (using up almost twice the number of kits than the previous day) when there were extreme shortages. Problem solving and prioritisation could've been better. | 6/29/2020 12:37 PM |
| 13 | good team work itu staff approachable | 6/25/2020 4:15 AM |
| 14 | I’m proud & grateful to be part of the proning team & despite getting the virus . I didn’t have a second thought to be part of the team again . I was able to use the experienced I had for myself too ! It was a relief ! Thsnk you Proning team ! Great team & I salute all those involved in this team . | 6/24/2020 2:56 PM |
| 15 | Please keep us all aware of any future opportunities as I personally and I'm sure the majority of the others on the team would be enthusiastic to continue to help in any capacity down the line. | 6/24/2020 1:38 PM |
| 16 | I felt very privileged to be part of the proning team. I enjoyed the team work aspect and knowing that I was doing a small role in helping those with Covid 19. It was a really positive feeling when a patient had improved enough to leave ICU. Maybe a little debrief at the end of each shift could have been useful. | 6/24/2020 11:47 AM |
| 17 | Proud to have been able to play a part in delivering care to those are most need. | 6/24/2020 7:49 AM |
| 18 | Good team, great spirit. | 6/23/2020 7:42 PM |
| 19 | As part of training most people trained how to prone however I never had to do this with a patient/team. Great feed back from people requiring the proning team. PPE not very good white gowns poor quality shrouds with the collar cut off, should not be used by anyone.  Coveralls very good. Well done though ITU staff great effort by those on the clinical areas. | 6/23/2020 7:03 PM |
| 20 | No | 6/23/2020 6:04 PM |

21

Amazing team, especially taking pressure off icu nurses, was a valuable experience, would

do it again

6

/23/2020 6:02 PM
