# Supplementary material for: Organisation and delivery of a dedicated multidisciplinary prone ventilation team in the intensive care unit: Strategies and lessons from COVID-19
Source: PLoS One. 2023 Dec 28;18(12):e0296379. doi: 10.1371/journal.pone.0296379 (PMC10754430; doi:10.1371/journal.pone.0296379)
Supplement: Attachment — Submitted filename: PONE-D-23-22349.docx [file pone.0296379.s005.docx]

PLOS ONE

Organisation and delivery of a dedicated multidisciplinary prone ventilation team in the intensive care unit: strategies and lessons from COVID-19

--Manuscript Draft--

| Manuscript Number: | PONE-D-23-22349 |
| --- | --- |
| Article Type: | Research Article |
| Full Title: | Organisation and delivery of a dedicated multidisciplinary prone ventilation team in the intensive care unit: strategies and lessons from COVID-19 |
| Short Title: | Delivery of a prone ventilation team |
| Corresponding Author: | Luke Bracegirdle  University Hospital Southampton NHS Foundation Trust UNITED KINGDOM |
| Keywords: | COVID 19  Prone position  Anaesthesia  Critical care  Intensive care  Mechanical ventilation Program development  Quality improvement |
| Abstract: | The global Coronavirus disease (COVID-19) pandemic necessitated a unique approach to healthcare delivery for critically ill patients. Prone ventilation emerged as a central management strategy for acute respiratory distress syndrome (ARDS) and acute hypoxic respiratory failure (AHRF), with early evidence suggesting its efficacy in severe COVID-19-related lung disease. This article presents a blueprint for establishing a dedicated 24-hour prone positioning team during the peak of the pandemic in Spring 2020. Our departmental experience was influenced by national guidelines from the Faculty of Intensive Care Medicine (FICM) and Intensive Care Society (ICS), as well as local expertise. The de-novo formation of this team effectively addressed the growing demand for multiple and prolonged prone manoeuvres among patients with COVID-19-related AHRF. Key considerations explored include multidisciplinary staff training, rapid assembly and deployment of clinical teams from various hospital departments, optimised rostering to ensure staff well-being, organisation of day-to-day prone positioning activities, and the importance of monitoring and continuous feedback for ongoing improvement. By establishing this dedicated prone positioning service with specifically trained staff, we significantly alleviated the workload and pressure on critical care teams. Ensuring the safe and timely implementation of prone manoeuvres positively influenced patient outcomes and enhanced staff well-being. |
| Order of Authors: | Luke Bracegirdle |
|  | Matthew Stubbs |
|  | Rezaur Rahman |
|  | Alexander I.R. Jackson |
|  | Helmi C. Burton-Papp |
|  | Robert Chambers |
|  | Sanjay Gupta |
|  | Michael P.W. Grocott |
|  | Ahilanandan Dushianthan |
| Additional Information: |  |
| Question | Response |

| Financial Disclosure  Enter a financial disclosure statement that describes the sources of funding for the work included in this submission. Review the [submission guidelines](http://journals.plos.org/plosone/s/submission-guidelines#loc-financial-disclosure-statement) for detailed requirements. View published research articles from [PLOS ONE](http://journals.plos.org/plosone/) for specific examples.  This statement is required for submission and will appear in the published article if the submission is accepted. Please make sure it is accurate. | The authors received no specific funding for this work. |
| --- | --- |
| Unfunded studies Enter: The author(s) received no specific funding for this work. Funded studies Enter a statement with the following details: - Initials of the authors who received each award - Grant numbers awarded to each author - The full name of each funder - URL of each funder website - Did the sponsors or funders play any role in the study design, data collection and analysis, decision to publish, or preparation of the manuscript? - NO - Include this sentence at the end of your statement: The funders had no role in study design, data collection and analysis, decision to publish, or preparation of the manuscript. - YES - Specify the role(s) played. |  |
| * typeset |  |
| Competing Interests  Use the instructions below to enter a competing interest statement for this submission. On behalf of all authors, disclose any [competing interests](http://journals.plos.org/plosone/s/competing-interests) that could be perceived to bias this work—acknowledging all financial support and any other relevant financial or nonfinancial competing interests.  This statement is required for submission | The authors have declared that no competing interests exist. |

| and will appear in the published article if the submission is accepted. Please make sure it is accurate and that any funding sources listed in your Funding Information later in the submission form are also declared in your Financial Disclosure statement.  View published research articles from [PLOS ONE](http://journals.plos.org/plosone/) for specific examples. |  |
| --- | --- |
| NO authors have competing interests Enter: The authors have declared that no competing interests exist. Authors with competing interests Enter competing interest details beginning with this statement: I have read the journal's policy and the authors of this manuscript have the following competing interests: [insert competing interests here] |  |
| * typeset |  |
| Ethics Statement  Enter an ethics statement for this submission. This statement is required if the study involved:   - Human participants - Human specimens or tissue - Vertebrate animals or cephalopods - Vertebrate embryos or tissues - Field research   Write "N/A" if the submission does not require an ethics statement.  General guidance is provided below. Consult the [submission guidelines](http://journals.plos.org/plosone/s/submission-guidelines#loc-guidelines-for-specific-study-types) for detailed instructions. Make sure that all information entered here is included in the Methods section of the manuscript. | This work was discussed with our institution’s research and development (R&D) team, and as a quality improvement project with findings that are completely anonymised, not generalisable and not transferable, and therefore do not qualify as research, explicit ethical approval was deemed not to be required.  Survey participant (adult healthcare professionals only) involvement was purely voluntary, and written information was provided at the start of the survey which was accessed by clicking on a link sent to participant’s email. |

Format for specific study types

Human Subject Research (involving human

participants and/or tissue)

Give the name of the institutional review

board or ethics committee that approved the

study

•

Include the approval number and/or a

statement indicating approval of this

research

•

Indicate the form of consent obtained

(

written/oral) or the reason that consent was

not obtained (e.g. the data were analyzed

anonymously)

•

Animal Research (involving vertebrate

animals, embryos or tissues)

Provide the name of the Institutional Animal

Care and Use Committee (IACUC) or other

relevant ethics board that reviewed the

study protocol, and indicate whether they

approved this research or granted a formal

waiver of ethical approval

•

Include an approval number if one was

obtained

•

If the study involved

non-human primates

,

add

additional details

about animal welfare

and steps taken to ameliorate suffering

•

If anesthesia, euthanasia, or any kind of

animal sacrifice is part of the study, include

briefly which substances and/or methods

were applied

•

Field Research

Include the following details if this study

involves the collection of plant, animal, or

other materials from a natural setting:

Field permit number

•

Name of the institution or relevant body that

granted permission

•

Data Availability

Authors are required to make all data

underlying the findings described fully

available, without restriction, and from the

time of publication. PLOS allows rare

exceptions to address legal and ethical

concerns. See the

[PLOS Data Polic](http://journals.plos.org/plosone/s/data-availability)

[y](http://journals.plos.org/plosone/s/data-availability)

and

[FA](http://journals.plos.org/plosone/s/data-availability#loc-faqs-for-data-policy)

[Q](http://journals.plos.org/plosone/s/data-availability#loc-faqs-for-data-policy)

for detailed information.

Yes - all data are fully available without restriction

| A Data Availability Statement describing where the data can be found is required at submission. Your answers to this question constitute the Data Availability Statement and will be published in the article, if accepted. |  |
| --- | --- |
| Important: Stating ‘data available on request from the author’ is not sufficient. If your data are only available upon request, select ‘No’ for the first question and explain your exceptional situation in the text box. |  |
| Do the authors confirm that all data underlying the findings described in their manuscript are fully available without restriction? |  |
| Describe where the data may be found in full sentences. If you are copying our sample text, replace any instances of XXX with the appropriate details.   - If the data are held or will be held in a public repository, include URLs, accession numbers or DOIs. If this information will only be available after acceptance, indicate this by ticking the box below. For example: All XXX files   are available from the XXX database (accession number(s) XXX, XXX.).   - If the data are all contained within the manuscript and/or Supporting Information files, enter the following: All relevant data are within the manuscript and its Supporting Information files. - If neither of these applies but you are able to provide details of access elsewhere, with or without limitations, please do so. For example:   Data cannot be shared publicly because of [XXX]. Data are available from the XXX Institutional Data Access / Ethics Committee (contact via XXX) for researchers who meet the criteria for access to confidential data.  The data underlying the results presented in the study are available from (include the name of the third party | All relevant data are within the manuscript |

| and contact information or URL).  • This text is appropriate if the data are owned by a third party and authors do not have permission to share the data.  * typeset |  |
| --- | --- |
| Additional data availability information: |  |

Manuscript	[Click here to access/download;Manuscript;Main document.docx](https://www.editorialmanager.com/pone/download.aspx?id=33717296&guid=8016e59c-89c9-4c2b-803e-00329f62a3c9&scheme=1)

**Organisation and delivery of a dedicated multidisciplinary prone ventilation team in**

# the intensive care unit: strategies and lessons from COVID-19

Luke Bracegirdle^1,2,3,4^, Matthew Stubbs^1^, Rezaur Rahman^1, 4^, Alexander I. R. Jackson^1,2,3,4^,

Helmi C. Burton-Papp^4^, Robert Chambers^4^, Sanjay Gupta^1,4^, Michael P.W. Grocott^1,2,3,4^,

Ahilanandan Dushianthan^2,3,4^

^1^Shackleton Department of Anaesthesia, University Hospital Southampton NHS Foundation Trust, Tremona Road, Southampton, SO16 6YD, UK

^2^Southampton NIHR Biomedical Research Centre, University Hospital Southampton NHS Foundation Trust, Tremona Road, Southampton. SO16 6YD, UK

^3^Faculty of Medicine, University of Southampton, University Hospital Southampton, Tremona Road, Southampton, SO16 6YD, UK

^4^General Intensive Care Unit, University Hospital Southampton NHS Foundation Trust, Tremona Road, Southampton. SO16 6YD, UK

# Corresponding Author

Dr Luke Bracegirdle^1,2,3,4^, NIHR Academic Clinical Fellow and Specialty Registrar

Anaesthetics and Intensive Care Medicine, Southampton General Hospital, University

Hospital Southampton NHS Foundation Trust, Tremona Road, Southampton. SO16 6YD, UK

Email: luke.bracegirdle@doctors.org.uk

# Keywords

COVID 19

Prone position

Critical care

Mechanical ventilation

Program development

**Declarations**

We have no conflict of interest or financial contributions to declare

# Background

In January 2020, the global pandemic of Coronavirus disease (COVID-19), caused by severe acute respiratory syndrome coronavirus 2 (SARS-CoV-2) viral infection, posed a significant threat to healthcare in the United Kingdom (UK). It became evident that a subset of COVID19 patients would experience critical illness, manifesting as acute respiratory distress syndrome (ARDS) and subsequent acute hypoxic respiratory failure (AHRF). The majority of these patients necessitated invasive mechanical ventilation, which has a substantial associated risk of mortality [1]. Initial reports from China and Italy highlighted the strain that COVID-19 placed on critical care resources, with mounting demand for mechanical ventilation and a substantial unmet need for additional qualified intensive care personnel.

Prone position ventilation has long been employed in managing ARDS patients and early evidence suggested potential benefits for COVID-19 patients with severe lung disease [2]. However, the process of proning critically ill, mechanically ventilated patients is timeconsuming and labour-intensive. Early in the COVID-19 pandemic, it became evident that existing intensive care staff were unable to perform the required multiple prone cycles without compromising bedside care. Consequently, we determined the need for a dedicated, specialised prone team to address the growing service demands, whilst ensuring patient and staff safety. By late March 2020, in response to the increasing number of patients in the General Intensive Care Unit (GICU) at University Hospital Southampton (UHS), a dedicated critical care prone team was established. Notably, early Intensive Care National Audit and Research Centre (ICNARC) data indicated UHS GICU as a positive (low mortality) outlier for risk-adjusted 28-day mortality from COVID-19 until August 31st, 2020 [3]. We attribute these positive outcomes, at least in part, to the swift implementation of the dedicated prone service.

This report outlines our experience in establishing a critical care prone service at UHS, sharing valuable insights and proposing a blueprint for its rapid reactivation in response to further

COVID-19 outbreaks or the emergence of novel respiratory illnesses in the future.

In the background please specify the major advers events that can occur during pronation and specify the incidence of adverse events (at least approximately) before and after the introduction of the team. In you experience which adverse events you hade the most? Extubations, impairment of gas exchange,hemodynamic instability, infected pressure ulcers.

# Methods

## Design and conception

The establishment of a prone positioning team to safely reposition mechanically ventilated patients in GICU required meticulous planning, preparation, and multidisciplinary collaboration. This work was discussed with our research and development department, and as a quality improvement project with participant involvement being purely voluntary, ethical approval was deemed not to be required. We assembled a team of senior clinical leaders and training personnel to oversee the development and implementation. Figure 1. outlines the key components of our local process for setting up the service.

Based on staffing requirements, we identified three categories of personnel crucial for successful prone manoeuvres.

## 1. Airway-Trained Personnel

These clinicians possess the skills to promptly identify and manage airway emergencies that may occur during prone positioning. They are prepared to perform rapid endotracheal intubation if accidental extubation of a critically hypoxic patient occurs. An experienced anaesthetist, intensivist, or advanced critical care practitioner

(ACCP) is suited for this role.

## 2. Team Members for Patient Turn

These individuals possess expertise in manual handling and are responsible for assisting in the physical turning of patients. Operating department practitioners (ODPs), theatre nurses, and practitioners from relevant surgical specialties (e.g., orthopaedics and neurosurgery) are well-suited for this task.

## 3. Team Members for Fine Positioning

This role involves precise positioning to prevent complications associated with the prone position, such as pressure injuries or mispositioned lines and circuits.

Healthcare workers from the aforementioned specialties commonly manage these

issues in theatre settings and are well-equipped to fulfil this responsibility.

# Strategy

## Training and Risk Mitigation

Based on simulation exercises, a concise training session of approximately 30-45 minutes was proposed, designed in accordance with the Faculty of Intensive Care Medicine (FICM) and Intensive Care Society (ICS) prone positioning guidelines [4]. Given the potential risks associated with prone positioning for both staff and patients, emphasis was placed on training, practice, and repetition. Staff undergoing training were required to demonstrate satisfactory manual handling competencies. The training sessions encompassed theoretical education, practical demonstrations, and hands-on practice using a high-fidelity setup with an intubated mannequin connected to a ventilator and various infusions and catheters as would be encountered in GICU.

## Resource Dissemination

To facilitate the widespread distribution of information and learning resources across different hospital departments, an online local application was developed (Appendix 1). This application provided easy access to essential documents such as the FICM and ICS prone position guidelines, training materials (e.g., prone positioning video demonstration), contact information, and a logbook. It was accessible from any browser or mobile device, independent of the staff intranet connection.

## Team Assembly (I suggest to move this paragraph before Resource dissemination)

Approximately 550 staff members from diverse clinical and non-clinical backgrounds received training. Recognising the uncertainties surrounding critical care services during the pandemic, a proactive decision was made to significantly scale up the number of staff trained in prone positioning. As knowledge about the virus and clinical outcomes evolved, more targeted training was provided, and ultimately, we deployed 78 staff members to full-time prone positioning teams.

## Training Delivery and Anaesthetic Support (I suggest to resume this paragraph with the training ad risk mitigation)

Training sessions were conducted by a dedicated nurse-led skills training team over several weeks, with multiple daily drop-in sessions. Separate sessions were held for approximately 60 anaesthetists, accounting for specific airway, ventilation, and cardiovascular considerations during prone positioning episodes. Simulation exercises guided the plans for ensuring 24-hour availability of experienced anaesthetists within the prone team. Additional tiers were added to the on-call anaesthetic rota to maintain safe anaesthetic coverage for other areas, responding to the increasing demand for anaesthetic support services. Recruitment of outpatient nursing staff and theatre teams further bolstered the team.

## Staffing Structure and Roster

Based on training exercises and early experiences with prone positioning in GICU, it was determined that a minimum of 5-6 members were required in each team. The standard team structure during the peak COVID-19 activity on GICU included one experienced anaesthetist or airway-trained clinician, four team members proficient in manual handling and positioning, and one bedside ITU nurse (typically the nurse assigned to care for that patient). Additional team members were occasionally necessary for repositioning obese or complex patients (such as those with multiple intercostal chest drains etc.).

Initially, prone teams were formed on an ad-hoc basis, utilising available anaesthetists supported by nursing, medical, and support staff. Ensuring comprehensive 24/7 coverage with a fully staffed prone positioning team required meticulous organisation, but increasing patient volume necessitated the development of a structured roster to sustainably perform over 40 prone manoeuvres every 24-hour period.

To evaluate the service, we sought regular feedback from the prone positioning team members. Feedback and learning points were disseminated on a regular basis to promote real-time service improvement.

# Results

The prone positioning service at UHS formally commended on March 26, 2020, running continuously for 8 weeks. Throughout this period, a total of 1208 prone and repositioning manoeuvres were performed, with a higher frequency of manoeuvres occurring outside regular working hours (Figure 2).

*Key Components of a Typical Prone Positioning Shift*

Figure 3. provides an illustration of the essential elements of a typical prone positioning shift.

## Importance of Team Leadership

Based on our experience, designating a team leader was crucial for effective coordination and communication with the GICU medical team. A simple whiteboard system facilitated patient tracking and safe handover. The team leader role was typically fulfilled by a senior nursing team member or an anaesthetist.

## Team Brief and Introductions

Given the multi-professional nature of the prone positioning teams and the likelihood of staff members not having worked together previously, we conducted a team brief before each shift. This included introductions with first names, roles, and a brief background experience description of each staff member. The team leader provided relevant information about the patients to be repositioned, including any important clinical details.

## Utilisation of Checklists

ICS and FICM guidelines formed the foundation for the locally adapted prone positioning protocols used at UHS. Checklists, encompassing indications, contraindications, and pre- and post-procedural checks ensured standardised and comprehensive practice. Printed and laminated checklists were readily available at each bed space, and electronic versions were accessible via the MicroGuide™ App and the local intranet (Appendix 2).

## Equipment and Drug Preparation

To familiarise new prone positioning team members with the critical care environment, a separate equipment and drug trolley was created for easy access to emergency supplies. This approach was derived from simulation-based training sessions and aligned with FICM guidance (4).

## Personnel Protective Equipment (PPE)

Following Public Health England (PHE) guidance [5], enhanced PPE was worn by staff to safeguard both themselves and patients. Considering the finite availability of PPE, we practiced the following; ensured comfort and a secure fit before entering clinical areas, cohorting patients to perform sequential prone positioning manoeuvres during a single PPE session, arranged escorts between ICU overflow areas to minimise PPE doffing, and scheduled prone positioning rounds to avoid coinciding with the clinical team’s PPE donning and doffing times.

## Documentation of Prone Positioning Episodes

We established a system to log prone positioning and repositioning events, which was essential for workload estimation, safety event reporting, and service improvement. The online logbook system was implemented, with completion responsibility typically assigned to the lead anaesthetist or team leader on each shift.

# Staff support

The ICU environment can present challenges for staff without prior exposure, leading to feelings of anxiety. Adequate preparation and support during training and deployment are crucial. Here we highlight the importance of familiarising new team members with the ICU environment, and provide recommendations to improve staff experience and wellbeing during mentally and physically demanding prone positioningshifts.

## Induction and preparation

To address the potential challenges faced by new or inexperienced team members, it is essential to provide comprehensive training. We recommend that new team members initially join the prone positioning team in a supernumerary role, allowing them to become familiar with the intensive care environment and its unique demands.

## Limiting Exposure

Working in PPE can be hot and uncomfortable, and prolonged periods in the ICU may increase the risk of cross infection. To improve staff experience and wellbeing, it is advised to limit staff exposure whenever possible. On our unit, in-hour anaesthetic prone positioning shifts were divided between two or three assigned anaesthetists, resulting in positive feedback and improved wellbeing among staff. Additionally, starting staggered prone positioning rounds with sufficient time to complete repositioning manoeuvres, as well as doffing well before handover helps avoid delays and reduces queues at doffing areas.

# Evaluation

Continuous auditing and evaluation of service development are essential for improving patient care. While quantifying the impact of individual prone positioning manoeuvres on individual patients is clearly beyond the scope of this report, tracking the overall quantity of manoeuvres performed provides a useful workload metric. We again emphasise the importance of using a logbook here.

In addition to volume metrics, understanding the lived experience of staff members who joined our prone positioning team is crucial. To gather valuable insights on service evaluation, we conducted an internal survey (see supplementary data) targeting 78 full-time team members.

The survey comprised 49 questions, and we received 61 responses from a diverse range of healthcare backgrounds.

## Demographic Profile (I suggest to put together demographic profile with the trainig paragraph and summarize them)

The majority of respondents were female (80%) and aged 25-54 years old (87%). Participants joined the team from various healthcare backgrounds, including medical, nursing, operating theatres, and research teams. 62% had not specifically volunteered for a role.

## Anxiety (I suggest to move this paragraph in the staff support section)

Most respondents reported feeling anxious, primarily related to infection risk and concern for spreading the infection to their families. There was also concern about returning to out-ofhours working patterns after working routine hours for some time

## Training

Formal training on prone positioning was provided to 92% of respondents, with 100% feeling adequately prepared to some extent. 84% had physically practiced on a person or mannequin, and 82% were shown and encouraged how to use our checklist. However, only 78% felt they received sufficient training, and they reported certain resources, such as using the prone positioning team web application, were less clearly emphasised with only 27% reporting they were shown how to use it. Potentially then, the quantity of prone positioning manoeuvres performed was an underestimate.

Of the respondents that had used the web application, 94% found it useful. 39% were aware of our prone positioning team training video, and all found it useful. Free text comments for training reported that some staff were not formally told of training sessions, and happened to find out from colleagues. Some thought that the mannequin size was not reflective of the real patients, and that the available checklists and videos were not highlighted during the face-toface training sessions. Of the respondents that underwent training, 57% went on the join the prone positioning team, but mentored or supernumerary shifts were available to only 17% of team members. Of those who did not have this opportunity, 70% reported that they would not have found it helpful anyway.

## Team Dynamics and Roles

Assigned roles varied, with 42% of respondents reporting they had undertaken the role of team leader during a shift. Teamwork within the prone positioning team was highly valued, with 93% reporting good teamwork. When asked specifically if there was good teamwork between the prone positioning team and the resident ICU team the majority (71%) agreed. Some reported mixed messages (??%) from ICU teams about which patients required prone positioning, and some raised concerns (??%) about perceived tension amongst teams and some obstructive comments about the use of muscle relaxants. There was a particularly strong feeling of teamwork between respondents and anaesthetists, with 98% reporting good teamwork. 95% reported always knowing who the team leader was and 70% reported they would know how to escalate any concern and importantly, felt their concerns would be taken seriously. 34% reported witnessing a clinical problem or emergency during a prone positioning procedure, with the majority (66%) of issues surrounding the ventilator or breathing circuit.

## Checklist Utilisation

The majority (93%) reported usually or always using the prone positioning checklist, with 91% finding it useful in preventing patient safety incidents. However, availability of the checklist was a concern, with only 65% reporting it was usually or always physically accessible. 9% reported that they witnessed incidents that they believed would have been prevented by the use of the checklist.

## PPE and safety

80% reported they had access to appropriate personal protective equipment (PPE) for every shift, but concerningly 62% reported they had spent longer than four hours in PPE at any one time. A small percentage (7%) tested positive for COVID-19, and 7% reported physical injuries. Comments included exacerbation of existing musculoskeletal symptoms, and sore skin from wearing PPE.

## Wellbeing

Reported number of prone positioning shifts undertaken varied, with 19% reporting they completed no shifts, and 2% reporting more than 40. They majority of people (65%) completed between one and 20 shifts. When asked about work intensity, the vast majority reported heavy (65%) or very heavy (7%). Breaks were usually or always sufficient for 84% of respondents, but sadly 11% reported negative psychological health effects, necessitating time off work for recovery.

## Overall Satisfaction

Despite the challenges faced, the majority (88%) felt proud to work on the prone positioning team, 82% felt valued, and 90% expressed willingness to join the team again.

# Discussion

Here we present the establishment of a dedicated prone positioning service in response to overwhelming demand during the COVID-19 pandemic. Our aim was to alleviate the workload and pressure on critical care clinical teams by rapidly training a large number of staff. We discuss the positive impact of safe and timely prone manoeuvres on benchmarked outcomes and staff well-being.

One notable aspect of our approach was the training of a significantly larger number of staff than those who ultimately joined the prone positioning team. This strategy aimed to create a larger pool of trained individuals, which could be considered beneficial. However, it also strained resources. Focusing teaching efforts on the smaller number of staff who joined the team could have further improved their confidence. While the methods we describe are generally transferable, it is important to note that our large, university teaching hospital may have greater access to resources than smaller centres. To gain insight into the lived human experience, we conducted a survey that received a high response rate. Although open surveys are susceptible to bias, the strong participation mitigates some of this risk. Nevertheless, it is worth acknowledging that the survey did not include responses from members of the clinical teams, which may limit our understanding of their perceptions.

Our findings align with previous work that emphasises the benefits of a multidisciplinary approach to prone positioning, which reduces complications in critically ill COVID-19 patients [6]. We also address barriers identified in another study, specifically focussing on knowledge and team culture [8]. While other centres have established prone positioning teams, they have not provided detail on training methods or explored the human impact through surveys [9], [10]. Some centres with smaller teams limited their service to daytime hours [11] [12], though interestingly their survey results echoed our findings, highlighting general satisfaction but with occasional power struggles.

To ensure the successful implementation or re-initiation of a prone positioning service, several factors should be considered. These include workload projections, the impact on other hospital areas, and collaboration among multiple teams and stakeholders. We recommend the formation of a dedicated "setup" team comprising senior personnel from relevant departments to provide effective leadership. Adequate training, including in-situ, high-fidelity simulation, is crucial, and maintaining a logbook for prone manoeuvres and complications facilitates risk identification, ongoing education, and team development. Prioritising rest and support for team members is essential for maintaining their well-being and performance. Regular feedback from the team members also provides valuable insights for improvement.

Although our work provides valuable insights, some questions remain unanswered. Future work could explore the direct impact of dedicated prone positioning teams on clinical outcomes. Additionally, incorporating the perspectives of clinical team members who were not surveyed would provide a more comprehensive understanding of the service's effectiveness. Evaluating the long-term sustainability and cost-effectiveness of prone positioning services across different healthcare settings is also necessary. Finally, exploring the potential benefits of advanced technologies, such as robotics or artificial intelligence, in prone positioning could open new avenues for future investigation.

# Conclusion

The COVID-19 pandemic was devastating and exhaustive to NHS staff, but has prompted innovative and collaborative approaches to healthcare delivery, yielding substantial improvements in patient outcomes. Here, we share our local experiences of initiating, organising, and implementing a dedicated prone ventilation service. Amid the peak COVID-19 activity in our ICU, we successfully conducted over 1200 individual prone positioning episodes, alleviated the burden on critical care staff and ensured the safe and timely provision of prone ventilation in our patients, contributing to excellent benchmarked outcomes. By sharing our experiences, we hope to inspire and encourage similar initiatives that enhance healthcare delivery and alleviate some of the challenges faced by critical care teams.

# References

1. Yang X, Yu Y, Xu J, Shu H, Xia J, Liu H, et al. Clinical course and outcomes of critically ill patients with SARS-CoV-2 pneumonia in Wuhan, China: a single-centered, retrospective, observational study. The Lancet Respiratory Medicine. 2020;8:475–81.
2. Pan C, Chen L, Lu C, Zhang W, Xia J-A, Sklar MC, et al. Lung Recruitability in COVID19–associated Acute Respiratory Distress Syndrome: A Single-Center Observational Study. Am J Respir Crit Care Med. 2020;201:1294–7.
3. Intensive Care National Audit & Research Centre. ICNARC, Reports, COVID-19 Report [Internet]. ICNARC. [cited 2023 Jun 21]. Available from: https://www.icnarc.org/ouraudit/audits/cmp/reports
4. The Faculty of Intensive Care Medicine and Intensive Care Society. Guidance For: Prone Positioning in Adult Critical Care [Internet]. 2019 [cited 2023 Jun 21]. Available from: https://ics.ac.uk/resource/prone-position-in-adult-critical-care.html
5. COVID-19: information and advice for health and care professionals [Internet]. GOV.UK. 2023 [cited 2023 Jun 21]. Available from: https://www.gov.uk/guidance/covid-19-informationand-advice-for-health-and-care-professionals
6. Ceruti S, Glotta A, Biggiogero M, Bona G, Saporito A, Faldarini N, et al. Multidisciplinary team approach in critically ill COVID-19 patients reduced pronation-related complications rate: A retrospective cohort study. Annals of Medicine and Surgery. 2021;70:102836.
7. Cotton S, Zawaydeh Q, LeBlanc S, Husain A, Malhotra A. Proning during covid-19: Challenges and solutions. Heart & Lung. 2020;49:686–7.
8. Klaiman T, Silvestri JA, Srinivasan T, Szymanski S, Tran T, Oredeko F, et al. Improving Prone Positioning for Severe Acute Respiratory Distress Syndrome during the COVID-19 Pandemic. An Implementation-Mapping Approach. Annals ATS. 2021;18:300–7.
9. Wells C, Zhang Z, Huelskamp S, Hughes E, Aguila D, Sevillano M, et al. Prone Team: A Large-Scale Prone Position Initiative During COVID-19 Pandemic. JONA: The Journal of Nursing Administration. 2021;51:E13.
10. Ng JA, Miccile LA, Iracheta C, Berndt C, Detwiller M, Yuse C, et al. Prone Positioning of Patients With Acute Respiratory Distress Syndrome Related to COVID-19: A RehabilitationBased Prone Team. Physical Therapy. 2020;100:1737–45.
11. O’Donoghue SC, Church M, Russell K, Gamboa KA, Hardman J, Sarge J, et al. Development, Implementation, and Impact of a Proning Team During the COVID-19 Intensive Care Unit Surge. Dimensions of Critical Care Nursing. 2021;40:321.
12. Short B, Parekh M, Ryan P, Chiu M, Fine C, Scala P, et al. Rapid implementation of a mobile prone team during the COVID-19 pandemic. Journal of Critical Care. 2020;60:230–4.

# Legends for Figures

Figure 1. The stages of development of the prone positioning team during the COVID-19

pandemic.

Figure 2. Prone position changes during peak COVID-19 activity on GICU.

Chart A shows the total number of prone position changes which occurred on a given day, during the peak period of activity. Chart B shows the relationship between total prone position changes and the times of day at which they occurred.

Figure 3. The key components of a typical prone positioning team shift. *Whiteboard* refers to a system usually involving a flipchart or wipeable surface used to track patient activity. *PPE* = Personal Protective Equipment. *Donning* refers to the process of applying PPE in a structured manner as directed by local guidance. *Base layer r*efers to the primary layer of PPE worn by a clinician. *Over-layer* refers to a disposable, second layer of PPE worn by a clinician when moving between patients in a cohorted area. *Doffing* refers to the process of removing PPE in a structured manner to reduce the risk of cross-infection.

# Contributions

LB: Manuscript preparation and review

MS: Study design, data collection, manuscript preparation

RR: Manuscript preparation, data collection

AIRJ: Data collection, analysis of Data

HCB-P: Data collection, analysis of Data

RC: Manuscript review

MPWG: Manuscript review

SG: Manuscript review

AD: Study design, analysis of Data, manuscript Review

**Competing interests**

The authors declare that they have no competing interests.

**Funding**

No funding received for this study

# Appendices

Appendix 1. Prone positioning app and logbook

Appendix 2. The General Intensive Care Unit Prone Ventilation Checklist

Appendix 1	[Click here to access/download;Figure;Appendix 1.tiff](https://www.editorialmanager.com/pone/download.aspx?id=33717340&guid=4bfc6926-f0da-46e3-8422-9e9fd243cbe8&scheme=1)

Appendix 2

What do you mean with PF > 20 (right column)	[Click here to access/download;Figure;Appendix 2.tiff](https://www.editorialmanager.com/pone/download.aspx?id=33717341&guid=91708f98-3fe7-473c-9b06-911749299587&scheme=1)
